# Trust in Physicians in the Context of HPV Vaccination of Children from the Perspective of Social Exchange Theory: A Representative Study of Polish Parents

**DOI:** 10.3390/vaccines11101618

**Published:** 2023-10-20

**Authors:** Tomasz Sobierajski, Piotr Rzymski, Ilona Małecka, Ewa Augustynowicz

**Affiliations:** 1 Center of Sociomedical Research, Faculty of Applied Social Sciences and Resocialization, University of Warsaw, 26/28 Krakowskie Przedmieście Str., 00-927 Warsaw, Poland; 2 Department of Environmental Medicine, Poznan University of Medical Sciences, 60-806 Poznań, Poland; rzymskipiotr@ump.edu.pl; 3 Integrated Science Association (ISA), Universal Scientific Education and Research Network (USERN), 60-806 Poznań, Poland; 4 Department of Preventive Medicine, Poznań University of Medical Sciences, 10 Fredry Str., 61-701 Poznań, Poland; ilona-malecka@wp.pl; 5 Department of Epidemiology of Infectious Diseases and Surveillance, National Institute of Public Health NIH—National Research Center, 24 Chocimska Str., 00-791 Warsaw, Poland; eaugustynowicz@pzh.gov.pl

**Keywords:** vaccine hesitancy, sociology, public health

## Abstract

The vaccination of children against human papillomavirus (HPV) effectively prevents HPV infection and HPV-related cancers in women and men. However, HPV vaccination programs are met with vaccine hesitancy, which varies between countries. The coverage in Poland is low, although introducing nationally funded HPV vaccination for girls aged 12–13 in mid-2023 may increase it. The uptake of the HPV vaccine in adolescents is highly affected by parental decisions, which in turn can be influenced by interactions with the physician. The present representative study aimed to analyze the acceptance of the HPV vaccine among Polish parents (*n* = 360) and the level of trust in HPV vaccination in the pediatrician/general practitioner who takes care of their children aged 9–15 years. The data were gathered in September 2022 using computer-assisted telephone interviews. Most surveyed parents reported trusting their child’s physician regarding vaccine recommendations (89.2%) and vaccinated their child with all or most of the vaccines recommended by a national vaccination guideline (94.7%). However, 13.3% declared themselves as moderate or strong vaccine opponents, a group characterized by high (83.4%) distrust in physicians. There was no difference in the awareness of HPV in groups varying in trusting the physicians, but parents who trusted them were more frequently aware of the HPV vaccine. Parental willingness to vaccinate their child against HPV was highly differentiated by the level of trust in the child’s physician. The results highlight that trust in physicians is a critical factor shaping decisions for children’s vaccination, stressing a continuous need to improve strategies to communicate with patients.

## 1. Introduction

As a major cause of cervical cancer (over 95% of cases), HPV remains a global issue [1]. HPV is also a significant cause of other genital cancers, including anal, vaginal, vulvar, penile, and oropharyngeal cancers [2]. Among the 690,000 HPV-attributable cancer cases identified annually, 83% are cervical cancer cases, of which 500,000 (72%) can be attributed to the high-risk HPV types 16 and 18 and 120,000 (17%) to HPV types 31, 33, 45, 52, and 58. Moreover, types 16 and 18 are responsible for nearly all HPV-related cancers diagnosed in men [3]. Therefore, HPV vaccination is essential to save lives because it can prevent HPV infections; the onset of moderate/severe cervical intraepithelial neoplasia, a precursor to invasive cervical cancer; and vulvar, vaginal, and anal cancer precursors and cancers [4,5,6,7,8]. HPV vaccination can also provide herd immunity, which means it can protect unvaccinated individuals by reducing the overall prevalence of HPV, as already evidenced in some communities [9,10,11].

There are currently three HPV vaccines available worldwide: (1) a bivalent vaccine against HPV types 16 and 18 [12,13]; (2) a quadrivalent vaccine against HPV types 6, 11, 16, and 18, of which the former two are responsible for nearly 90% of genital wart cases [14,15,16,17]; and (3) a nonavalent vaccine against HPV types 6, 11, 16, 18, 31, 33, 45, 52, and 58, infection with which is related to nearly 90% of all cervical cancer cases [18]. All three vaccines are administered as a series of two or three doses, depending on the age and local recommendations [19,20,21]. 

According to the World Health Organization (WHO), as of the first quarter of 2023, 125 countries have introduced HPV vaccination in their national immunization programs [22]. The highest coverage rates for HPV vaccination are found in high-income countries such as Australia, Canada, the United States, and some European countries [23,24]. The European Union (EU) has recommended HPV vaccination for girls aged 9–14 since 2008, but each member state implements it according to its own national program [25]. In 2020, the European Centre for Disease Prevention and Control recommended implementing a gender-neutral approach to HPV vaccination, providing the vaccine to both boys and girls to achieve the highest protection against HPV transmission and HPV-related diseases [26]. This approach has been adopted by some European countries in recent years [27]. 

In Poland, vaccination has been recommended for girls aged 12–13 years since 2008, with a catch-up program offered to girls aged 14–18 years from 2008 to 2012. However, for a long time Poland remained the only country in the European Union not to introduce reimbursed HPV vaccinations in the national immunization program. HPV vaccinations were exclusively local and scarcely funded [28] until mid-2023, when nationally funded, voluntary HPV vaccination was offered to girls and boys aged 12–13 years (but in other age groups remained available only commercially) [29]. The vaccination rates in Poland remain low compared with other EU countries—as of 2021, 25% of girls and 10% of boys aged 9–14 received a vaccine. From the point of view of public health policy, the level of HPV vaccination must increase in Poland, particularly if one considers that 16.9 million women are at risk of cervical cancer, with annual morbidity and mortality of over 3800 and 2100, respectively [30,31]. In addition, there has been a recent increase in the incidence of oropharyngeal cancers, particularly in men [32,33,34,35]. HPV type 16 is one of the causative agents of these cancers, and the increase has been attributed to changes in sexual behaviors and a lack of HPV vaccination in the population of Polish men.

Various factors can influence the parental decision to reject HPV vaccination of children, including perceived insufficient or inadequate information and knowledge about the vaccine, fear of side effects, mistrust in health authorities, doubts about the vaccine’s effectiveness, and low perceived risk of HPV infection or cervical cancer [36,37,38,39]. One of the key elements in influencing parents’ attitudes and their decision to vaccinate their children against HPV is a high level of trust in physicians, as they are the primary sources of information and advice about vaccines. Patients who trust their physicians are more likely to have fewer concerns about vaccine safety, higher confidence in their efficiency, and follow the vaccination recommendations [40,41,42,43]. According to the Social Exchange Theory (SET), trust and cooperation are based on exchanging resources and expecting mutual benefits [44]. Patients are more likely to trust in and comply with their physician’s recommendations if they perceive these individuals as providing high-quality care and that the benefits of vaccination outweigh the costs or risks. Achieving it requires effective communication, respect for patient autonomy, and a collaborative approach.

Taking into account the risks arising from the currently low level of HPV vaccination in Poland, the opportunity to increase it due to the recent reimbursement vaccination program in adolescents, and the importance of physician–patient trust in the context of vaccination, the present study evaluated parents’ attitudes towards their children’s HPV vaccination with a particular focus on the level of trust in vaccination in the pediatrician/general practitioner who takes care of their children aged 9–15 years. Based on a literature review, an analysis of the studies conducted, and social and medical knowledge, we hypothesized that those parents who declare greater trust in the physician caring for their child declare greater willingness to vaccinate them against HPV.

## 2. Materials and Methods

### 2.1. Design of the Study and Study Sample

The present survey was conducted in September 2022 and involved 360 parents of children aged 9–15 years. A stratified random sampling technique was employed to ensure the sample’s representativeness [45]. We prioritized the child’s place of residence, gender of the child, and gender and age of the parent bracket based on data provided by the Central Statistical Office in 2021. To achieve an accurate representation, we divided the respondents into urban and rural areas as well as the 16 Polish provinces. 

The data were gathered using computer-assisted telephone interviews (CATIs) conducted by trained interviewers who asked the questions from the questionnaire and entered the responses using specialized software. The software ensured the coherence of the answers and set control questions to maintain the survey’s quality. Furthermore, the responses were added to the database in real-time to minimize the risk of data loss. 

### 2.2. The Questionnaire

The present study utilized a survey questionnaire tailored explicitly for research purposes and developed by the authors, who have extensive expertise in sociological, methodological, and medical domains. To ensure the questionnaire’s accuracy and appropriateness for the study, an interdisciplinary team conducted an ad hoc validation process. The questionnaire comprised 18 questions, comprising nine metric and nine factual questions. All the questions were of the single-choice type, with some being dichotomous. The metric parameters included gender, age, education level, number of children, and gender of the youngest child. The factual questions assessed the participants’ attitude towards vaccination, level of trust in vaccine recommendations given by their child’s doctor, and willingness to vaccinate their child against HPV. The selection and phrasing of questions were based on our previous experiences in analyzing sociological and sociomedical issues in the context of vaccination [41,46,47,48,49]. 

To guarantee the validity and reliability of the survey questionnaire, a pilot study was conducted involving 12 parents of children aged 9–15. It allowed us to assess the questionnaire’s conceptual validity, methodological soundness, and structural correctness. The results of the pilot study were not included in the main study but were helpful in refining the questionnaire for subsequent administration to a larger sample size. 

### 2.3. Statistical Analysis

The demographic variables of the surveyed group were presented with descriptive statistics. The outcome variable was focused on the HPV vaccine, and the relationship between variables was evaluated by employing the Chi-squared test. All statistical analyses were performed using IBM SPSS Statistics v. 29.0.0.0 (IBM Corp., Armonk, NY, USA). A *p*-value of less than 0.05 was deemed statistically significant in all analyses. 

### 2.4. Ethical Considerations

The survey was carried out by the Biostat, a research unit with Research and Development Center status registered under the Minister of Entrepreneurship and Technology in Poland. This registration guarantees that the survey was conducted ethically and complies with international ethical requirements for quantitative research. The study followed Polish law regarding protecting the subjects’ data and followed the ethical guidelines for implementing sociomedical research. The study did not have the nature of a clinical trial, and no sensitive data were collected during the study, so the consent of the ethics committee, according to Polish law, was not required. Each subject was informed about the purpose of the study and gave informed consent to participate. The study was anonymous and confidential. The study’s results were analyzed collectively, making it impossible to identify a specific person from the study’s results. 

## 3. Results

### 3.1. Sociodemographic Characteristics of Respondents

The sociodemographic characteristics of the surveyed parents of children aged 9–15 years are summarized in Table 1. Two-thirds of the respondents were mothers and over 50% were aged ≥40 years and had completed at least secondary education. Most of the surveyed parents had at least two children, and the distribution of their gender was similar (Table 1).

### 3.2. Parental Level of Trust in Doctors and General Attitudes to Vaccines

**Figure 1:**
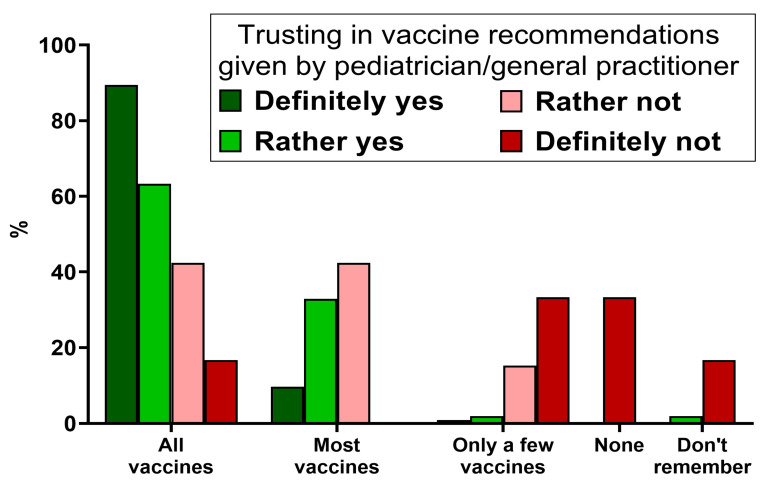
The distribution of answers regarding their child’s vaccination status in relation to the parental level of trust in vaccination recommendations given by the child’s pediatrician/general practitioner (*N* = 360).

Most respondents reported trusting their child’s doctor regarding their vaccination recommendations (89.2%) and vaccinated their child with all or most of the vaccines recommended by a national vaccination guideline (94.7%). The lower the trust in the doctor, the statistically significantly lower the percentage of parents who vaccinated their child with the available vaccines, according to the current vaccination calendar (*p* < 0.001) (Figure 1).

Based on the provided declarations, the studied parents were divided into four groups depending on their general attitude toward vaccines: (i) strong supporters (34.7%), (ii) moderate supporters (51.9%), (iii) moderate opponents (10.0%), and (iv) strong opponents (3.3%).

**Figure 2:**
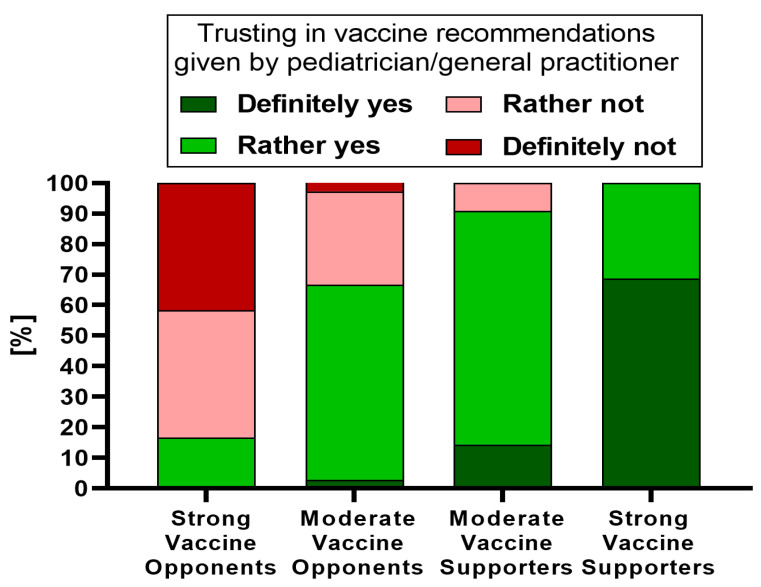
The distribution of answers regarding parental trust in vaccination recommendations given by the child’s pediatrician/general practitioner in relation to general parental attitudes towards vaccination (*N* = 360).

Parents who declared themselves strong supporters of vaccination were statistically significantly more likely to trust their doctor regarding their child’s vaccinations compared with those who classified themselves as strong opponents (*p* < 0.001) (Figure 1). The difference was highly contrasting, as over half of the strong opponents (83.4%) declared that they definitely did not or would rather not trust their child’s doctor regarding the vaccine recommendations, while among strong vaccine supporters, this level of trust reached 100% (Figure 2).

### 3.3. Parental Level of Trust in Doctor and Attitude towards HPV and HPV Vaccines

**Figure 3:**
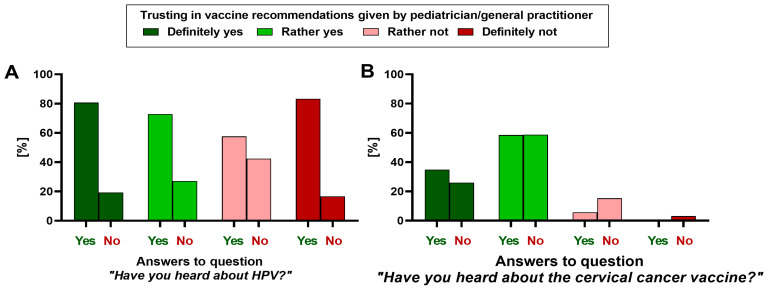
The distribution of parental answers regarding (**A**) awareness of HPV and (**B**) the cervical cancer vaccine in relation to the parental level of trust in the vaccination recommendations given by the child’s pediatrician/general practitioner (*N* = 360).

The frequency of parents who heard about HPV did not differ significantly between groups with varying levels of trust in the vaccination recommendations given by their child’s doctor (*p* = 0.052) (Figure 3A). However, parents willing to trust the doctor were more frequently aware of the existence of the cervical cancer vaccine (*p* = 0.005) (Figure 3B).

**Figure 4:**
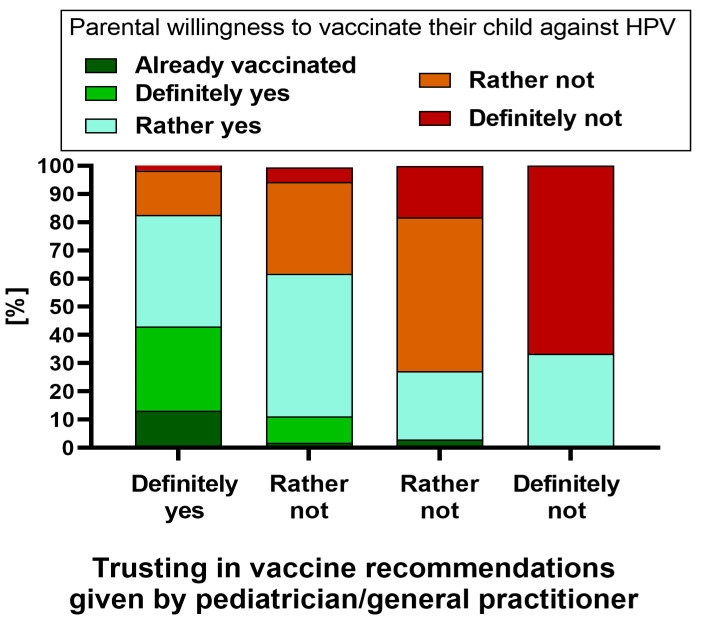
The parental willingness (*N* = 360) to vaccinate their child against HPV in relation to the parental level of trust in the vaccination recommendations given by the child’s pediatrician/general practitioner.

Among those who declared they definitely trusted their doctor for vaccine recommendations, 13.2% of children have already been vaccinated against HPV, and seven out of ten people intend to vaccinate their child against HPV (*p* < 0.001) (Figure 4).

## 4. Discussion

The present study shows that the parental level of trust in a child’s physician is associated with better adherence to national guidelines for childhood vaccination, better awareness about HPV and the HPV vaccine, and willingness to vaccinate a child against HPV. The results indicate that trust is a critical factor in healthcare, especially for children. Studies show that patients who trust their healthcare providers are more likely to follow medical advice, have better outcomes, and have higher satisfaction with their care [50,51,52]. Parents’ trust in their pediatrician concerning their child’s health has special significance, which is interestingly captured with the SET theoretical framework, according to which interpersonal relationships, including doctor–patient relationships, should be considered in terms of partisan cost–benefit analyses of the action taken and the commitment to it. Parents often perform cost–benefit analyses in great depth regarding a child’s health. In addition, the cost–benefit analysis is particularly complex in the context of vaccinations, which have a preventive form, being compensators rather than direct rewards that, for example, cure disease or relieve pain. Parents and guardians rely on pediatricians to provide accurate information about vaccinations and make recommendations that align with the best interests of their child’s health. From the perspective of this theory, parents’ trust in the doctor regarding vaccinations is a very strong “currency.” The metanalysis by Brewer et al. examined the relationship between risk perception and vaccination behavior across a number of studies. The authors used SET to explain why individuals might choose not to get vaccinated even if they perceive a high risk of contracting a particular disease [53]. Indeed, much depends on the quality of the vaccination messages delivered through different platforms and the reception of these messages [54]. In a study by Eller et al., mothers who had a low degree of trust in their child’s doctor were much more likely to use other sources of information about vaccination, which may have led to a more frequent decision not to vaccinate their child [55]. Therefore, it is crucial to utilize multiple media platforms to deliver consistent, evidence-based messages.

Although vaccines are medical products, the decision to receive them is a social phenomenon that numerous factors, including trust in healthcare providers, can influence. The parents surveyed in the present study declared a relatively high trust in their children’s physicians, and most of them adhere to national childhood vaccination guidelines. These results indicate that there is a good groundswell of the wide acceptance of HPV vaccination among Polish parents. However, one should note that refusals of compulsory vaccines for children doubled in Poland over the last five years and have risen 21-fold since 2010 [56]. Moreover, research shows more than one-third of young (15–39 years) Poles do not support mandatory vaccinations for children, and 75% are against the penalization of vaccine refusal [57]. Such attitudes may eventually deter others from vaccinating their children with mandatory and recommended vaccines, including the HPV vaccine. The present study shows that over 10% of the surveyed parents declared themselves moderate or strong opponents of vaccination, a group among which most individuals declared a low level of trust in the vaccine recommendations given by their children’s physicians. It can be hypothesized that vaccine hesitancy and distrust in physicians may be promoted during the COVID-19 pandemic due to unseen misinformation campaigns that employed various communication platforms, inducing online social media, that facilitate the rapid spread of fake news [58,59,60,61]. This may now have an impact on the intake of HPV vaccines in Poland as well as other vaccines to be recommended in the future.

Our study shows that those parents who declare greater trust in the doctor caring for their child in the context of vaccination declare a greater willingness to vaccinate their child against HPV. The review by Harrington et al. indicates that trust in the doctor to vaccinate a child against HPV is very important, but other functional (low levels of trust in the pharmaceutical industry) and social (associating HPV vaccination with sexual initiation) factors that may—despite high trust in the doctor—influence the ultimate failure to vaccinate a child cannot be ignored [62]. Moreover, a study by Liddon et al. conducted on a sample of 1268 parents of children between the ages of 11 and 17 indicated that a high level of parental trust in the pediatrician has a powerful influence on the willingness to vaccinate a child against HPV [63]. At the same time, it is important to note that trust is a complex and multi-dimensional concept that various factors, such as communication style, cultural beliefs, and personal experiences, can influence [64,65,66]. Therefore, children’s physicians must engage in active communication on cervical cancer risk and preventive measures with parents/caregivers. It highlights that physicians require professional training to understand parental fears and sources of vaccine hesitancy, build trust, and deliver accurate and honest vaccine messages regarding the risk and benefits [67,68]. It is recommended that messages on HPV vaccines should focus on cancer prevention rather than sexual transmission, highlight the risks and costs of HPV infection, indicate the widespread nature of HPV and the potential for its non-sexual transmission, and appeal to the moral responsibility of parents to protect their children from severe diseases such as cancer [69].

However, one should note that ensuring appropriate messaging for HPV vaccination is not limited to the physician–patient interaction. The studies show that the attitudes of healthcare providers toward the HPV vaccine can be influenced by their trust in the stakeholders involved in designing and implementing the HPV vaccination strategy [70]. Therefore, pediatricians should be reinforced by the health system with training to increase their knowledge of immunization, which influences their cognitive attitudes and recommendations of vaccination to parents of children [71]. This has already been shown with COVID-19 vaccines, where healthcare workers who underwent dedicated, specifically designed workshops on vaccine communication were more confident in recommending them to their patients; therefore, they should receive similar support regarding HPV vaccinations [72,73].

### Study Limitations

We wish to stress study limitations that pertain to the methodology employed. Respondents may exhibit a Halo Effect when using the CATI technique to conduct surveys, which causes them to provide expected responses instead of expressing their factual thoughts and knowledge. Such an effect can particularly occur when assessing attitudes toward sensitive subjects such as child vaccination. A limited sample size was included in the study, if compared with the total population size of Poland. The findings might be reliable; however, the representativeness might be biased. One should also note that the present research was conducted at a time when national HPV vaccinations were about to be introduced in Poland, and, therefore, they may serve as a reference point for further studies on attitudes during the ongoing vaccination campaign.

## 5. Conclusions

This study highlights that parental trust in the medical doctors taking care of their child is critical in the decision to vaccinate them against HPV. There is a groundswell for good acceptance of HPV vaccination among parents in Poland, but an effort must be undertaken to ensure good-quality and honest communication with those who oppose vaccines, declare distrust in healthcare providers, and are not willing to vaccinate their children against HPV.

## Figures and Tables

**Table 1:** Sociodemographic characteristics of respondents (*N* = 360).

Parameter	% (*n*)
**Parent’s gender**	
Female	63.1 (227)
Male	36.9 (133)
**Age (years)**	
<30	8.1 (29)
30–39	33.3 (120)
40–49	50.3 (181)
>49	8.3 (30)
**Education level**	
Primary	15.0 (54)
Secondary	43.6 (157)
Tertiary	41.4 (149)
**Number of children**	
1	25.3 (91)
2	49.4 (178)
3	17.5 (63)
≥4	7.8 (28)
**Gender of the youngest child**	
Female	48.9 (176)
Male	51.1 (184)

## Data Availability

The raw data supporting the conclusions of this article will be made available by the authors upon request.
